# Integrated Genomic and Proteomic Analysis Reveals T‐B Lymphocyte Signatures in the MYCN Driven “Immune Desert” of Specific Neuroblastoma Subtypes

**DOI:** 10.1002/cns.71054

**Published:** 2026-09-01

**Authors:** Jia‐Jian Hu, Wen‐Juan Kang, Jia‐Tong Hu, Ye‐Xiong Li, Feng‐Ju Song, Min Deng

**Affiliations:** ^1^ State Key Laboratory of Molecular Oncology and Department of Radiation Oncology National Cancer Center/National Clinical Research Center for Cancer/Cancer Hospital, Chinese Academy of Medical Sciences (CAMS) and Peking Union Medical College (PUMC) Beijing China; ^2^ Acupuncture Department Guang'anmen Hospital, China Academy of Chinese Medical Sciences Beijing China; ^3^ Department of Epidemiology and Biostatistics, Key Laboratory of Molecular Cancer Epidemiology in Tianjin, Tianjin's Clinical Research Center for Cancer, National Clinical Research Center for Cancer Tianjin Medical University Cancer Institute & Hospital Tianjin China

**Keywords:** B lymphocytes, immune desert, MYCN, neuroblastoma, single‐cell RNA sequencing, T lymphocytes, transcription factors, tumor microenvironment

## Abstract

**Aims:**

This study aims to systematically dissect how MYCN amplification shapes the immunosuppressive tumor microenvironment (TME) in high‐risk neuroblastoma, elucidating key mechanisms underlying immune evasion.

**Methods:**

We performed an integrated multi‐omics analysis of bulk RNA‐seq (*n* = 721), single‐cell RNA‐seq (*n* = 9), proteomic data (*n* = 49) and spatial transcriptomics (Visium, with external validation in melanoma). Analyses included unsupervised clustering, cell–cell communication inference, transcriptional regulatory network reconstruction, and spatial proximity assessment to map the immune landscape.

**Results:**

A distinct molecular subtype (Class C), defined by MYCN amplification and poor prognosis, exhibited a comprehensive “immune desert” phenotype characterized by low immune scores and minimal leukocyte infiltration. Single‐cell analysis confirmed significant depletion of T and B lymphocytes within the Class C TME. Dysregulated transcriptional networks were identified, including upregulation of REL and EOMES in T cells—with EOMES potentially driving exhaustion via regulation of Transient Receptor Potential (TRP) genes, and REL inhibition enhancing cytotoxic function in vitro. A unique immunosuppressive B‐cell subset (B7) engaged in enhanced crosstalk with exhausted T cells and harbored a MYC‐centered network linked to cell cycle dysregulation and poor survival. Spatial transcriptomics revealed significant proximity between B7‐active regions and Treg/exhaustion‐enriched areas, externally validated in melanoma. Proteomic data validated elevated REL expression in MYCN‐amplified tumors.

**Conclusion:**

This work delineates the immunosuppressive architecture of MYCN‐driven neuroblastoma, revealing novel regulatory nodes within specific lymphocyte compartments. Integrating single‐cell, spatial, and proteomic evidence, we propose REL inhibition as a therapeutic candidate, the EOMES/TRP axis as a bioinformatically supported hypothesis, and the B7/MYC hub as a hypothesis supported by transcriptomic and spatial evidence.

## Graphical abstract

This study identifies a high‐risk neuroblastoma subtype defined by MYCN amplification and an “immune desert” tumor microenvironment. We uncover novel lymphocyte‐specific immunosuppressive networks, including T cell dysfunction and a MYC‐driven B cell hub, revealing potential therapeutic targets.

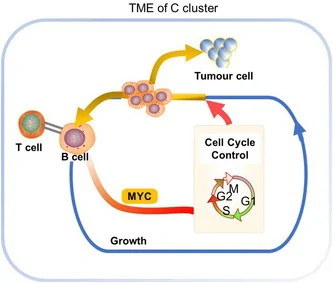

## 1 Introduction

Neuroblastoma (NB) is a heterogeneous pediatric tumor originating from neural crest cells [1]. Its tumor microenvironment (TME) is a complex ecosystem of neoplastic, stromal, and immune cells, along with signaling molecules and extracellular matrix, which interact to regulate tumor progression and immune evasion [2]. Understanding the roles of anti‐tumor immune cells is crucial for developing new therapies [3]. The anti‐tumor immune response is conceptualized as the Cancer‐Immunity Cycle (CIC), a sequential process from antigen release to T cell‐mediated killing [4]. The balance of this cycle determines whether the TME is immunologically active (“hot”) or suppressed (“cold”) [5].

Advances in single‐cell and bulk RNA sequencing now allow systematic profiling of the TME and key immune checkpoints like PD‐L1 [6, 7], driving immunotherapy development [8]. Enhanced bioinformatics tools enable deeper analysis of immune cell states and the CIC [9]. Building on these advances, our study integrates bulk RNA‐seq (*n* = 721), single‐cell RNA‐seq (*n* = 9), spatial transcriptomics, and proteomic data (*n* = 49) to dissect the NB TME across multiple resolution scales [10]. Using bioinformatic tools including Scissor, CellCall, and spatial proximity analysis, we aim to characterize the immunosuppressive TME in high‐risk, MYCN‐driven NB and elucidate the underlying transcriptional and cellular mechanisms, with particular focus on T‐B lymphocyte crosstalk and its spatial organization within the tissue microenvironment.

## 2 Materials and Methods

### 2.1 Materials and Data Sources

#### 2.1.1 Bulk RNAseq Data

Dataset GSE49710 was downloaded from the NCBI Gene Expression Omnibus (GEO), comprising gene expression profiles from 498 NB samples profiled on the Agilent02382 Human Microarray 44k platform (GPL16876) [11, 12, 13]. Designated NB01 in this study, it includes detailed clinicopathological information such as International Neuroblastoma Staging System (INSS) stage, age, sex, MYCN amplification status, survival outcome, overall survival (OS), and event free survival (EFS).

Dataset EMTAB8248 was downloaded from the EMBLEuropean Bioinformatics Institute (EMBLEBI) ArrayExpress database, comprising 223 NB samples profiled on the ag44kcwolf platform [14]. Designated NB02, its clinical information includes MYCN status, INSS stage, sex, disease progression status, survival outcome, and OS.

#### 2.1.2 10× Genomics scRNAseq Data

Dataset GSE147766 was obtained from GEO, involving 19 NB samples sequenced on the Illumina NextSeq 500 platform (GPL18573) [15, 16]. Clinical metadata included age, risk stratification, and primary or relapse status. Based on the study design, we selected 9 cases meeting all three criteria‐age > 18 months, high risk, and primary tumor‐for subsequent in‐depth analysis.

#### 2.1.3 Mass Spectrometry Proteomics Data

Dataset PXD018300 was acquired from the ProteomeXchange Consortium. Clinical information included MYCN score and INSS stage. Samples lacking followup data, expression data, or those with documented interventional treatments were excluded [16, 17].

#### 2.1.4 Spatial Transcriptomics Data

Our study analyzed two datasets from the NCBI Gene Expression Omnibus (GEO) database: GSE250636 and GSE309269, both of which are spatial transcriptomics data [18, 19].

All these data and information used were derived from public, freely accessible database management systems, which have undergone ethical review by their respective sources, thus obviating the need for further approval from our institutional ethics committee. No written consent has been obtained from the patients as there is no patient‐identifiable data included.

### 2.2 Research Methods

#### 2.2.1 Software and Data Preprocessing

This study utilized R (v4.1.1) for core analysis, Perseus (v2.0.6.0) for proteomics, and Cytoscape (v3.8.2) for network visualization. Data from public repositories (e.g., GEO, ProteomeXchange) were processed as follows: Bulk RNA‐seq data (721 samples) were log2‐transformed, normalized, and merged. scRNA‐seq data (9 samples) were quality‐filtered (cells with > 200 genes and < 5% mitochondrial content), integrated using CCA/MNN to correct batch effects, and normalized with LogNormalize in Seurat. Proteomics data were processed in Perseus by filtering contaminants, log2‐transforming, and imputing missing values [20, 21].

#### 2.2.2 Dimensionality Reduction and Clustering of Bulk RNA‐Seq Data

The merged bulk RNA‐seq data from 721 samples were analyzed through unsupervised clustering. This process began with the identification of highly variable genes (HVGs) using Seurat's “FindVariableFeatures” function. Principal Component Analysis (PCA) was performed for dimensionality reduction. Subsequently, a cell relationship graph was constructed based on key principal components using K‐nearest neighbor (KNN) and shared nearest neighbor (SNN) algorithms. Samples were then partitioned into molecular subtypes via community detection with the Louvain algorithm. Finally, Uniform Manifold Approximation and Projection (UMAP) was applied for nonlinear dimensionality reduction and visualization of the results.

#### 2.2.3 Calculation of Tissue Immune Status

Tumor immune status was assessed using a multi‐tiered computational approach. The ESTIMATE algorithm (ssGSEA‐based) was applied to compute immune/stromal/ESTIMATE scores and tumor purity [22, 23]. Immune cell infiltration was quantified via ssGSEA using a 28‐cell‐type gene set from MSigDB. Activity scores for immune‐related molecules (chemokines, MHC molecules, etc.) were derived from TISIDB gene lists. Finally, Cancer‐Immunity Cycle activity was evaluated by scoring its seven steps with gene sets from the TIP web server.

#### 2.2.4 Immune and Clinical Characterization of New Subtypes and HALLMARK Enrichment Analysis

HALLMARK pathway analysis characterized the new subtypes. Gene Set Variation Analysis (GSVA) was applied to expression data from 721 samples using gene sets from GSEA, followed by differential analysis (limma, |*t*| > 2). Clinicopathological data, immune indicators, and differential pathways were integrated, normalized, and visualized to elucidate subtype relationships [24, 25, 26].

#### 2.2.5 Dimensionality Reduction and Clustering of NB scRNA‐Seq Data

The integrated single‐cell data from nine samples (32,363 cells) were processed using the standard Seurat pipeline. Highly variable genes (HVGs) were identified (“FindVariableFeatures,” “vst” method) and scaled (“ScaleData”). Principal Component Analysis (PCA) was performed on scaled HVGs. The top 15 principal components, selected based on standard metrics, were used for downstream analysis. Cell clustering was performed by constructing a k‐nearest neighbor (KNN) graph in this PCA space (“FindNeighbors”) and applying the Louvain algorithm (“FindClusters,” resolution = 2). Results were visualized using t‐SNE and UMAP for nonlinear dimensionality reduction [27, 28, 29].

#### 2.2.6 Identification of Tumor Cells and Annotation of Cell Clusters in scRNA‐Seq Data

A multi‐step approach was used to distinguish tumor from normal cells. First, copy number variation (CNV) was inferred using the CopyKAT algorithm to classify cells as diploid (normal) or aneuploid (tumor) based on scRNA‐seq expression profiles [30, 31, 32]. Automated cell type annotation was then performed with the SingleR package by correlating cell transcriptomes against reference datasets [33, 34, 35]. Final annotations were determined by integrating Seurat clustering results, CopyKAT classifications, and SingleR labels [36, 37, 38, 39, 40, 41, 42, 43].

#### 2.2.7 Application of the Scissor Tool

The Scissor algorithm was used to link bulk RNA‐seq defined subtypes with scRNA‐seq defined cell subpopulations [44, 45, 46]. It requires the single‐cell expression matrix, the bulk expression matrix, and corresponding bulk phenotypic labels: Class C versus NOC (Non‐Class C). Scissor calculates a Pearson correlation matrix between each cell and each bulk sample, then builds a regression model using this correlation against the phenotype. This model assigns a regression coefficient (*β*) to each cell, where a high positive β indicates a stronger association with the target phenotype (e.g., Class C).

#### 2.2.8 Subpopulation Analysis of T and B Lymphocytes

Based on Scissor results highlighting T and B lymphocytes as key discriminators, these cells were extracted and reclustered for deep subpopulation analysis. Cell subsets were identified by integrating marker gene information from the CellMarker and PanglaoDB databases with literature review. Lineage identity was assigned via canonical markers, while subclusters were denoted by their representative differentially expressed genes (DEGs) combined with lineage. Functional annotations were assigned by examining marker expression patterns within clusters using visualizations like violin plots [47, 48].

#### 2.2.9 Differential Analysis of T Lymphocyte Subpopulations

To investigate T lymphocyte functional differences between Class C and NOC TME, differential expression analysis was performed using the scCODE R package. scCODE robustly identifies Differentially Expressed Genes (DEGs) by integrating multiple data filtering and differential analysis methods, selecting the top‐performing strategies based on concordance metrics [28, 49, 50]. The resulting DEGs (filtered at adjusted *p*‐value < 0.05 and |log2FoldChange| > 1) were subsequently subjected to GO and pathway enrichment analysis using the ClueGO plugin in Cytoscape to construct functional networks [51, 52, 53].

#### 2.2.10 Evaluation of REL Inhibitor IT‐901 Effects on Cytotoxic Function

Human peripheral blood mononuclear cells (PBMCs) from healthy donors were pre‐treated with the REL inhibitor IT‐901 (3 μM) or DMSO control in IL‐2‐supplemented medium. The cytotoxicity of these PBMCs was assessed against CFSE‐labeled tumor cells in a co‐culture assay. Specific killing was quantified by flow cytometry as the percentage of CFSE + PI+ target cells, with a target cell‐only control included to account for spontaneous death.

#### 2.2.11 Virtual REL Knockout in Tumor‐Infiltrating Treg Cells

Treg cells were extracted from a neuroblastoma scRNA‐seq dataset. REL‐positive cells were sampled, and the top 2000 variable genes plus REL were retained after filtering. Virtual knockout of REL was performed using scTenifoldKnk (3 replicates, K = 2). Differentially regulated genes were identified at adjusted *p* < 0.05 and |*Z*| > 2.

#### 2.2.12 Differential Analysis of B Lymphocyte Subpopulations

The analytical method was identical to that used for T lymphocyte subpopulations. The scCODE package identified DEGs between Class C and NOC B cells, followed by HALLMARK pathway enrichment analysis on the DEGs to uncover underlying biological functional changes.

#### 2.2.13 Analysis of Key Transcription Factors

To decipher the gene regulatory networks underlying the observed differential expression, transcription factor analysis was performed. First, key transcription factors (TFs) were predicted using the IRegulon Cytoscape plugin, analyzing the top 30 upregulated DEGs from T/B cells with a threshold of NES ≥ 6 [54]. Subsequently, a TF‐microRNA‐mRNA regulatory network was constructed by querying the miRWalk database with the predicted TFs and their target genes, followed by functional enrichment analysis of the associated miRNAs using miEAA. Finally, the JASPAR database was used to predict and validate potential TF binding sites in target gene promoters [55, 56, 57]. Protein–protein interaction (PPI) networks of candidate transcription factors were constructed using the STRING database and visualized in Cytoscape to identify hub regulatory nodes associated with B7‐cell functional programs.

#### 2.2.14 Cell–Cell Communication Analysis of T‐B Lymphocyte Subpopulations

Intercellular communication between T and B lymphocyte subpopulations was analyzed using the CellCall R package [58]. This method integrates ligand‐receptor interaction data from multiple databases and constructs ligand‐receptor‐transcription factor (LR‐TF) regulatory axes by incorporating downstream TFs from KEGG pathways [59]. The workflow involves comparing ligand‐receptor interaction strengths between Class C and NOC samples and assessing downstream pathway activity to infer functionally significant communication events within the TME.

#### 2.2.15 Spatial Validation on Visium Sections From Neuroblastoma and Melanoma

Visium datasets from NB (GSE309269, cervical lymph node metastasis) and melanoma (GSE250636) were processed with Seurat. Module scores for B7 core, B‐cell, T‐cell, and Treg/exhaustion signatures were calculated. High‐score spots were defined by quantile thresholds. Euclidean distances between B7‐high and Treg/exhaustion‐high, B7‐high and other T‐cell‐high, and other B‐cell‐high and Treg/exhaustion‐high spots were computed and compared using Wilcoxon tests with Benjamini–Hochberg adjustment.

### 2.3 Statistical Analysis

Group comparisons (e.g., ESTIMATE scores) were performed using the Wilcoxon rank‐sum test. Survival analysis was conducted using the Kaplan–Meier method, with differences between groups compared by the log‐rank test. Correlation analysis utilized Spearman's correlation. A *p* value < 0.05 was considered statistically significant. All analyses were performed within the R language environment.

## 3 Results

### 3.1 General Characteristics of the Included NB Samples

**FIGURE 1:**
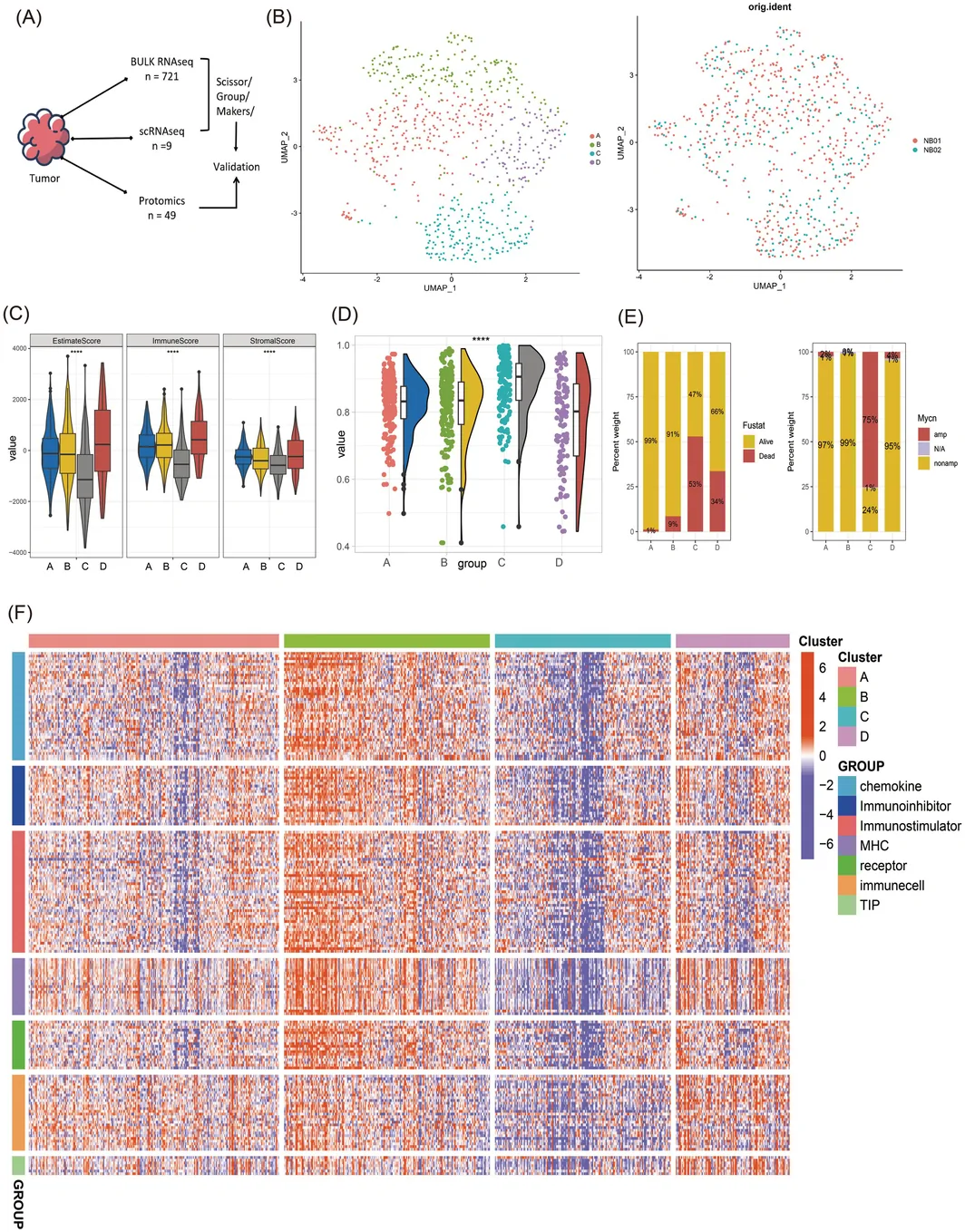
Identification and immune characterization of the MYCN‐driven “immune desert” subtype (Class C) in neuroblastoma. (A) Overview of the integrated multi‐omics cohorts. (B) UMAP plot of bulk RNA‐seq data from 721 samples defining four molecular subtypes (A–D). (C) Comparison of ESTIMATE algorithm‐derived scores (Immune, Stromal, ESTIMATE) and tumor purity across the four subtypes. (D) Violin plots comparing tumor purity between Class C and Non‐Class C (NOC) groups. (E) Enrichment of clinical features across the new subtypes. Stacked bar plots show the proportions of survival outcomes (Dead/Alive) and MYCN amplification status (amp, amplified; nonamp, non‐amplified) within each subtype (A–D). (F) Comprehensive immune landscape analysis across subtypes. Heatmaps display the relative enrichment scores, calculated using ssGSEA, for: 28 immune cell types (gene set from GSEA), activity of the 7‐step Cancer‐Immunity Cycle (gene set from TIP), and expression levels of immune‐related molecules including chemokines, MHC molecules, immunostimulators, immunosuppressors, and receptors (gene lists from TISIDB). Statistical significance: ****p < 0.0001. amp, amplified; NOC, Non‐Class C; nonamp, non‐amplified.

Bulk RNAseq After rigorous quality control and filtering, 721 NB samples (NB01 + NB02) were included. INSS stage distribution Stage 1 (*n* = 150), Stage 2 (*n* = 117), Stage 3 (*n* = 99), Stage 4 (*n* = 272), Stage 4 s (*n* = 83). MYCN status Amplified (amp, *n* = 138), Not Amplified (noamp, *n* = 577), NA (*n* = 6). Survival outcome Deceased (*n* = 147), Alive (*n* = 574). scRNAseq nine samples meeting the criteria (age > 18 months, high risk, primary) were selected, yielding 32,363 high‐quality cells for subsequent analysis. Proteomics 49 samples. MYCN amplified (*n* = 38), not amplified (*n* = 11). INSS stage Stage 1 (*n* = 10), Stage 2 (*n* = 5), Stage 3 (*n* = 2), Stage 4 (*n* = 23), Stage 4s (*n* = 9) (Figure 1A).

### 3.2 Unsupervised Clustering Defines Four Molecular Subtypes

Unsupervised clustering of bulk RNAseq data from 721 samples revealed clear separation into four molecular subtypes, designated A, B, C, and D. Sample counts were Class A (*n* = 242), Class B (*n* = 199), Class C (*n* = 170), Class D (*n* = 110). This classification successfully integrated samples from two distinct datasets (NB01, NB02), demonstrating its robustness (Figure 1B).

### 3.3 Immune Landscape of New Subtypes Reveals Clinical Class C as an “Immune Desert”

Analysis using the ESTIMATE algorithm revealed that Class C samples had significantly lower ESTIMATE, Immune, and Stromal scores, alongside higher tumor purity, compared to other subtypes (*p* < 0.0001; Figure 1C,D). Class C was strongly associated with aggressive clinical features, being highly enriched for MYCN amplification (accounting for 92.75% of all amplified cases) and fatal outcomes (61.22% of all deaths; Figure 1E). ssGSEA further demonstrated that Class C exhibited comprehensively lower scores across multiple immune dimensions, including immune cell infiltration, Cancer‐Immunity Cycle activity, and expression of immune‐related molecules, characterizing it as an “immune desert” (Figure 1F). HALLMARK pathway analysis indicated Class C was enriched for processes related to proliferation, DNA repair, and metabolism, such as MYC targets and oxidative phosphorylation (Figure S1).

### 3.4 scRNAseq Data Quality Control, Integration, and Cell Atlas Construction

Following calculation of quality control (QC) metrics for each cell and scatter plots validating, scRNA‐seq data from nine samples were analyzed (Figure S2A,B). Highly variable genes (HVGs) were identified, with 1500 selected for downstream analysis (Figure S3A). After Seurat‐based integration, tumor cells were inferred using CopyKAT. Automated annotation (SingleR) and manual curation classified 32,363 cells into 10 major types (Figure S3B): T cells (11,216), B cells (3445), macrophages (803), monocytes (3207), NK cells (2993), smooth muscle cells (1219), neuronal cells (900), tissue stem cells (1500), endothelial cells (1329), and tumor cells (5751).

### 3.5 Scissor Projection Reveals Decreased T/B Lymphocyte Proportions in Class C TME

The Scissor algorithm was employed to project the bulk RNA‐seq derived phenotypes (Class C vs. NOC) onto the single‐cell data. This analysis revealed significant heterogeneity in TME cellular composition (Figure S3C). Compared to NOC, the Class C “immune desert” exhibited a decreased proportion of total immune cells and a specific reduction in T and B lymphocytes, alongside an increase in stromal cells. This established T and B lymphocytes as key distinguishing immune components, warranting their deep subpopulation analysis.

### 3.6 T Cell Subpopulations Exhibit Functional Alterations in Class C

**FIGURE 2:**
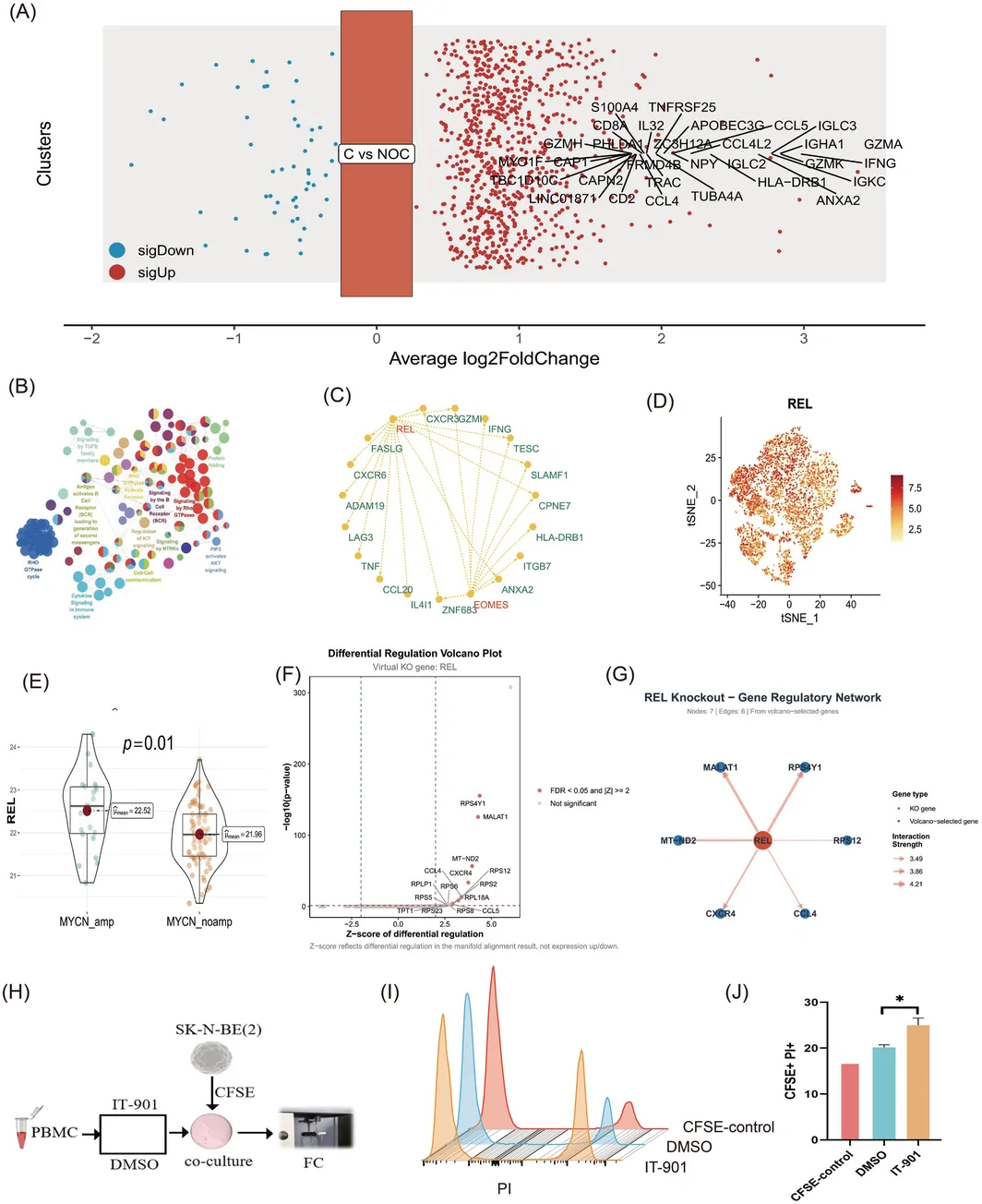
Molecular alterations and key transcription factors in T lymphocytes within the Class C TME. (A) Heatmap of the top 30 upregulated DEGs in Class C T cells. (B) ClueGO network of enriched pathways (e.g., Rho GTPase cycle) among T cell DEGs. (C) IRegulon‐predicted TF regulatory network highlighting REL and EOMES. (D) Expression of REL across T cell subpopulations. (E) Proteomic validation of higher REL protein expression in MYCN‐amplified samples. (F) Volcano plot showing genes differentially regulated after REL virtual knockout. Red points indicate significant genes (FDR < 0.05, |*Z*‐score| ≥ 2), with representative targets labeled. (G) Regulatory network of volcano‐selected genes after REL knockout. REL acts as the central node connected to six affected genes; edge thickness indicates interaction strength. (H) Schematic of the in vitro cytotoxicity assay workflow. (I) Representative flow cytometry plots of tumor cell killing by PBMCs pre‐treated with DMSO or the REL inhibitor IT‐901. (J) Quantification of specific tumor cell killing from three independent experiments. (Statistical significance in E and J: *p < 0.05). DEG, differentially expressed gene; TF, transcription factor; TME, tumor microenvironment.

The extracted T cells (*n* = 11,216) were reclustered, identifying 11 distinct subpopulations (Figure S3D), including regulatory T cells (Tregs), and exhausted T cell (Tex) subsets, among others. Differential analysis of T lymphocytes in the Class C TME using scCODE identified 847 DEGs. After filtering (adj. *p*‐value < 0.05, |log2FC| > 1), 288 significant DEGs remained (Figure 2A). Pathway enrichment analysis (ClueGO) of these DEGs revealed enhanced activity of the Rho GTPase cycle, chemokine signaling, and B cell receptor signaling pathways in Class C T cells (Figure 2B).

### 3.7 Regulatory Roles of Key Transcription Factors REL and EOMES in T Lymphocytes

IRegulon transcription factor prediction (NES > 6) on the top 30 upregulated DEGs in T cells identified two key TFs REL and EOMES (Figure 2C). They regulate multiple genes involved in T cell activation, migration, and exhaustion, including IL4I1, CCL20, CXCR3, LAG3, IFNG, and FASLG. Expression analysis showed that REL was broadly upregulated across most T cell subpopulations, whereas EOMES was highly expressed primarily in Tregs (Figure 2D, Figure S4A). Furthermore, proteomic data validated (Figure 2E) significantly higher REL protein expression in the MYCN‐amplified group (*p* < 0.05). To evaluate the regulatory role of REL, we performed virtual knockout analysis of REL. The differential regulation volcano plot showed that multiple genes were significantly affected after REL knockout, with most significant genes displaying positive differential regulation *Z*‐scores. Using the criteria FDR < 0.05 and |*Z*‐score| ≥ 2, genes such as MALAT1, RPS4Y1, MT‐ND2, CXCR4, CCL4, and RPS12 showed strong differential regulation signals. Notably, the *Z*‐score reflects differential regulation inferred from manifold alignment, rather than conventional expression up‐ or down‐regulation (Figure 2F). A gene regulatory network was then constructed using volcano‐selected genes. REL was positioned as the central node and directly connected to MALAT1, RPS4Y1, MT‐ND2, CXCR4, CCL4, and RPS12, suggesting that REL may act as a key regulator influencing immune chemotaxis‐related, mitochondrial, ribosomal, and transcription‐associated genes (Figure 2G).

Pre‐treatment with the REL inhibitor IT‐901 significantly enhanced PBMC cytotoxicity against tumor cells (procedure, Figure 2H). Flow cytometry showed a higher mean percentage of dead target cells in the IT‐901 group (25.0%) versus control (20.1%) (spontaneous death: 17.0%). Specific killing was calculated, revealing IT‐901 treatment augmented cytotoxicity approximately 1.45‐fold (*p* < 0.05; Figure 2I,J), demonstrating its potent enhancement of anti‐tumor activity.

For EOMES, 31 associated microRNAs were predicted via miRWalk, and a ceRNA network was constructed (Figure S4B). Pathway analysis of these miRNAs in miEAA primarily showed enrichment for “Inflammatory mediator regulation of TRP channels” (Figure S4C). Given the established link between TRP channels and inflammation/immune regulation, we further analyzed the relationship between TRP family genes and T cell exhaustion scores, finding a significant positive correlation for most members (Figure S4D). JASPAR database predictions indicated highscoring binding sites for EOMES in the promoter regions of major TRP family genes such as TRPA1, TRPC1, TRPM1, and TRPV1 (Figure S4E, Table 1), suggesting EOMES may directly regulate TRP channel gene expression, thereby positively modulating T lymphocyte exhaustion.

**TABLE 1:** Predicts potential binding sequences in promoter regions with a score threshold of 0.8 or higher on the coding strand.

Name	Score	Relative score	Sequence ID	Start	End	Strand	Predicted sequence
MA0800.1.EOMES	8.991804	0.845655338	TRPA1	1412	1424	+	AAAGTGTGAGGGG
MA0800.1.EOMES	7.402508	0.819661984	TRPA1	1281	1293	−	TAAGTGTCAATCG
MA0800.1.EOMES	6.8880796	0.811248378	TRPA1	1684	1696	−	GGGGTGTAAAGAG
MA0800.1.EOMES	7.6137676	0.823117193	TRPC1	1772	1784	+	TGGGTGTGGAAAG
MA0800.1.EOMES	7.4767623	0.820876436	TRPC1	1075	1087	+	AAACTGTGATATA
MA0800.1.EOMES	7.447478	0.82039748	TRPC1	563	575	−	ATGGCGTGATCTT
MA0800.1.EOMES	6.5437555	0.805616868	TRPC1	1262	1274	−	TGAGTGAGAACAT
MA0800.1.EOMES	9.604359	0.855673827	TRPM1	1203	1215	+	GGGGTGTGAGAGA
MA0800.1.EOMES	9.081268	0.847118548	TRPM1	258	270	−	GATGTGTGAGGAG
MA0800.1.EOMES	10.969353	0.87799866	TRPV1	658	670	−	CAGGTGAGAAATG
MA0800.1.EOMES	9.982725	0.861862109	TRPV1	649	661	−	AATGTGTGACGCT
MA0800.1.EOMES	7.2903304	0.817827294	TRPV1	245	257	−	TAGGCGTGAGCCA

### 3.8 B Lymphocyte Subpopulation Identification and Their Cell Cycle Dysregulation in Class C

**FIGURE 3:**
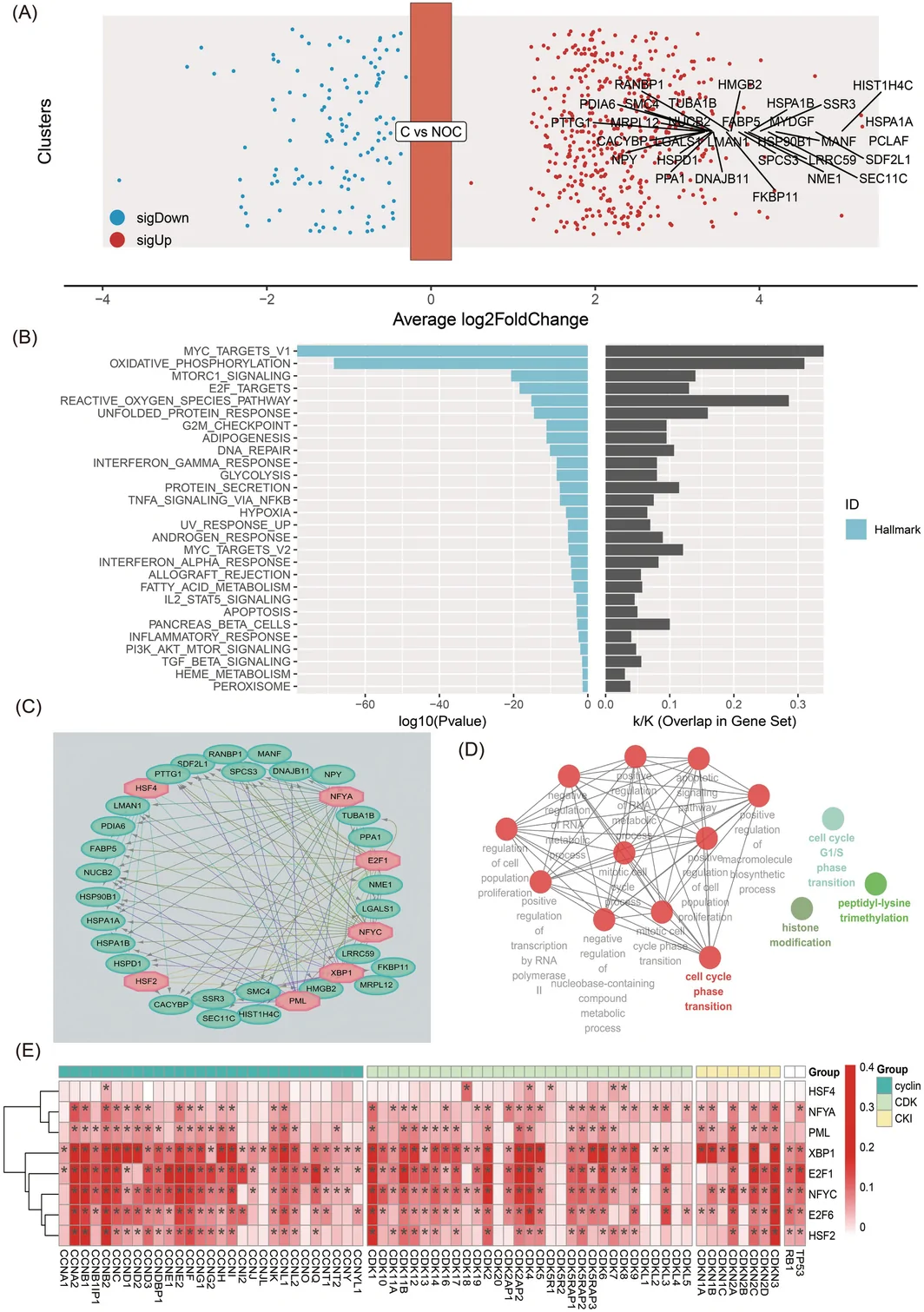
Dysregulated cell cycle pathways and associated transcription factors in B lymphocytes of Class C. (A) Volcano plot of DEGs in B lymphocytes. (B) Top enriched HALLMARK pathways among upregulated DEGs. (C) Diagram of the key TFs (e.g., NFYA, E2F1) predicted to regulate top genes. (D) ClueGO network showing the involvement of the 8 TFs in cell cycle regulation. (E) Correlation heatmap between the 8 key TFs and core cell cycle regulators. DEG, differentially expressed gene; TF, transcription factor; TME, tumor microenvironment.

B cells (*n* = 3445) were extracted and reclustered, identifying 10 B cell subpopulations (Figure S3E). Differential analysis between Class C and NOC B cells identified 561 Differentially Expressed Genes (DEGs). Following filtering, 505 significant DEGs remained (Figure 3A). HALLMARK pathway enrichment analysis revealed these DEGs were enriched in MYC Targets V1, Oxidative Phosphorylation, and mTORC1 signaling (Figure 3B). IRegulon prediction on the top 30 upregulated genes identified eight key transcription factors (TFs): NFYA, NFYC, E2F1, XBP1, HSF4, HSF2, PML, and E2F6 (overlaps E2F1, omitted) (Figure 3C). ClueGO analysis indicated these TFs are significantly involved in cell cycle processes, including phase transition and proliferation regulation (Figure 3D). Their expression showed widespread correlation with core cell cycle regulators (Figure 3E).

### 3.9 Shift Towards Immunosuppressive Communication Between T‐B Lymphocyte Subpopulations in Class C

**FIGURE 4:**
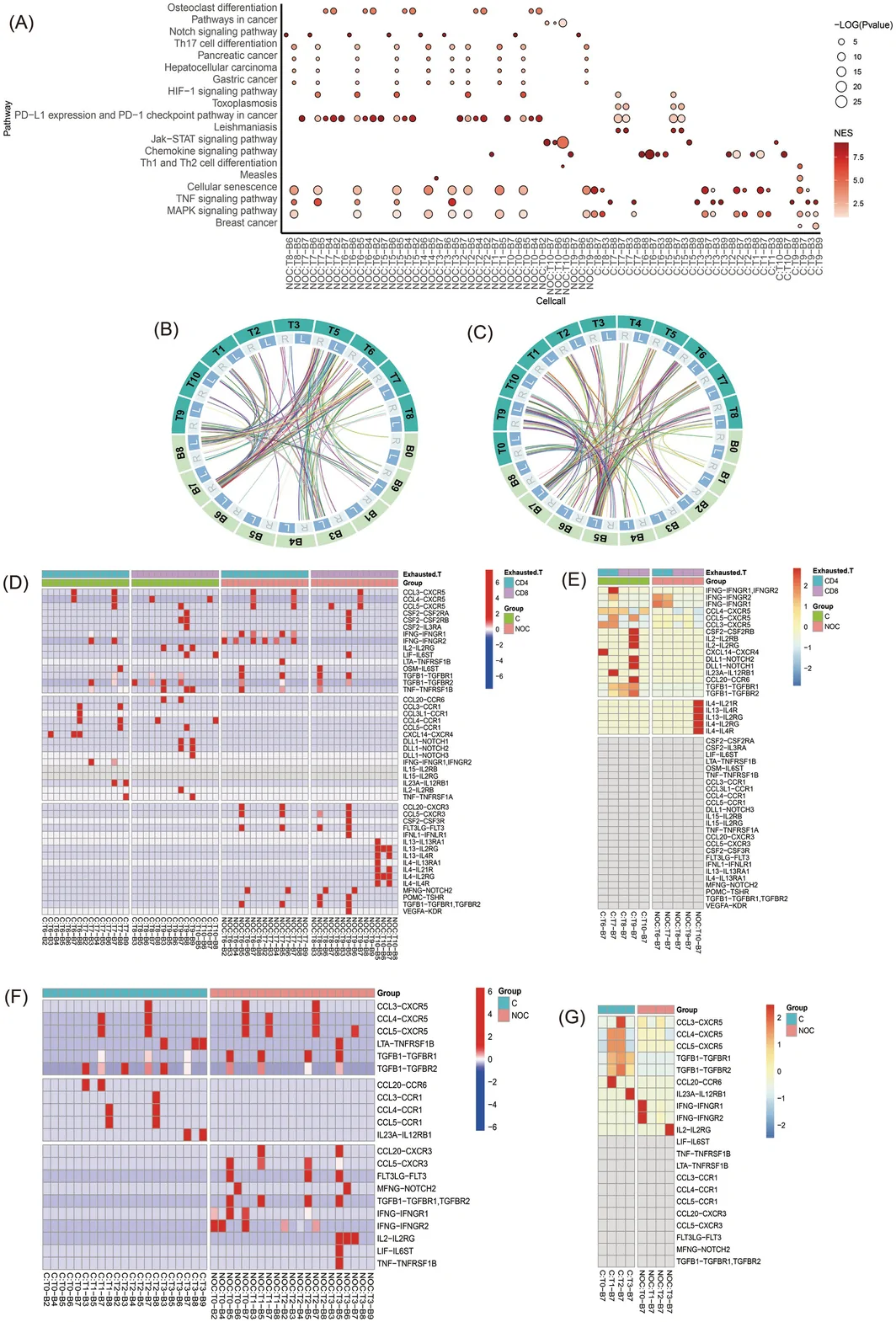
Altered immunosuppressive cell–cell communication between T and B lymphocytes in Class C. (A) Heatmap comparing the activity of key communication pathways (e.g., TNF, Chemokine) initiated from specific T cell subsets (exhausted CD8+ T, exhausted CD4+ T, Tregs) towards all B cell subsets in Class C versus NOC. (B, C) Circos plots depicting the overall cell–cell communication networks between T and B lymphocyte subpopulations in (B) Class C and (C) NOC, respectively. (D) Analysis of significant ligand‐receptor pairs mediating communication between exhausted T cell subsets and all B cell subsets. (E) Detailed analysis of ligand‐receptor interactions specifically between exhausted T cell subsets and the B7 subpopulation (as receptor). (F) Analysis of significant ligand‐receptor pairs mediating communication between regulatory T cell (Treg) subsets and all B cell subsets. (G) Detailed analysis of ligand‐receptor interactions specifically between regulatory T cell subsets and the B7 subpopulation (as receptor).

Cell–cell communication analysis was performed on all T and B lymphocyte subpopulations using CellCall. Pathway level compared to NOC, in Class C (Figure 4A). Exhausted CD8^+^ T cells (T8—T10) as ligands showed significantly enriched signaling pathways, for example, TNF, while exhausted CD4^+^ T cells (T6–T7) and Tregs (T0–T4) showed significantly enriched signaling pathways, for example, chemokine. Notably, the B cell subpopulation B7 appeared as a receptor in all these significantly differential communication pathways, suggesting B7 is a key hub receiving inhibitory T cell signals. The B7 subset was characterized by high expression of RGS13, LRMP, MARCKSL1, MKI67, PCLAF, HMCES, PTTG1, HMGA1, PKM, ACTG1, HMGB2, HMGB1, HMGN2, H2AFZ, HIST1H4C, TUBB, STMN1, and TUBA1B. Network level Cell communication network diagrams showed (Figure 4B,C) that B7 shifted from being a primary signal “sender” in NOC to a major signal “receiver” in Class C, with a marked increase in the total number of signals received.

Ligand‐receptor pair level detailed analysis of communication between exhausted T cells (Figure 4D) and Tregs (Figure 4F) with B cells revealed significant enhancement in Class C of chemokine interactions such as CCL3/4/5‐CCR1/5 and CCL20‐CCR6, as well as signals like IL15/23 and TNF superfamily members. Focusing specifically on Treg communication with B7, interactions were significantly increased in Class C. We provided a detailed view of the specific ligand‐receptor interactions between exhausted T cells, Treg subsets and the B7 subpopulation as receptor (Figure 4E,G).

**FIGURE 5:**
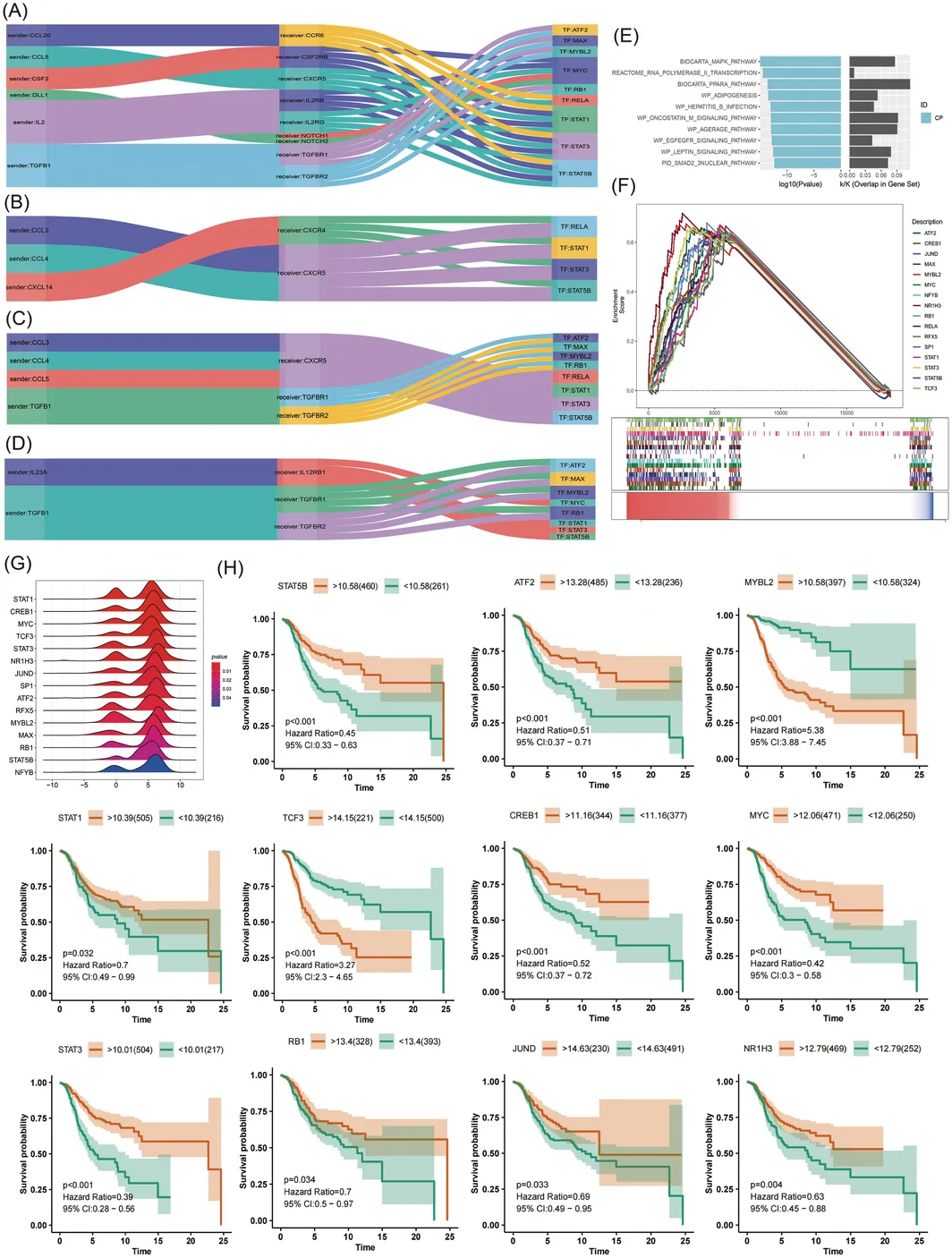
The LR‐TF axis reveals an activated MYC‐centered network in the B7 subpopulation. (A–D) Ligand‐Receptor‐Transcription Factor (LR‐TF) axis analysis for key interactions involving the B7 subpopulation as the common receiver: (A) exhausted CD8+ T cells‐B7‐TF axis, (B) exhausted CD4+ T cells‐B7‐TF axis, (C) Treg‐B7‐TF axis (T2), (D) Treg‐B7‐TF axis (T3). (E) Pathway analysis of the downstream transcription factors (TFs) identified via the LR‐TF axis, showing their significant association with cancer progression. (F) The activation states of all key TFs within B7 cells. (G) Expression levels (reflecting activation) of representative downstream target genes of the key TFs in the B7 subpopulation. (H) Kaplan–Meier survival analysis demonstrating the prognostic impact of the key transcription factors on NB patient overall survival. LR‐TF, ligand‐receptor‐transcription factor; Treg, regulatory T cell.

Downstream TF analysis of the key T‐B cell communication pairs (T9B7, T6B7, T2B7, T3B7) (Figure 5A–D) identified 16 activated key TFs within B7 cells ATF2, CREB1, JUND, MAX, MYC, MYCL2, NFYB, NR1H3, RB1, RELA, RFX5, SP1, STAT1, STAT3, STAT5B, TCF3. Pathway analysis indicated most of these TFs participate in cancer progression (Figure 5E), and they were all in an activated state within B7 cells, with their downstream target genes also generally upregulated (Figure 5F,G). Survival analysis linked 11 of the 16 TFs to overall survival (Figure 5H). High expression of MYBL2 and TCF3 was significantly associated with poor prognosis. GSVA indicated that samples with high MYBL2/TCF3 expression upregulated cell cycle‐related pathways (e.g., DNA replication; Figure S5).

**FIGURE 6:**
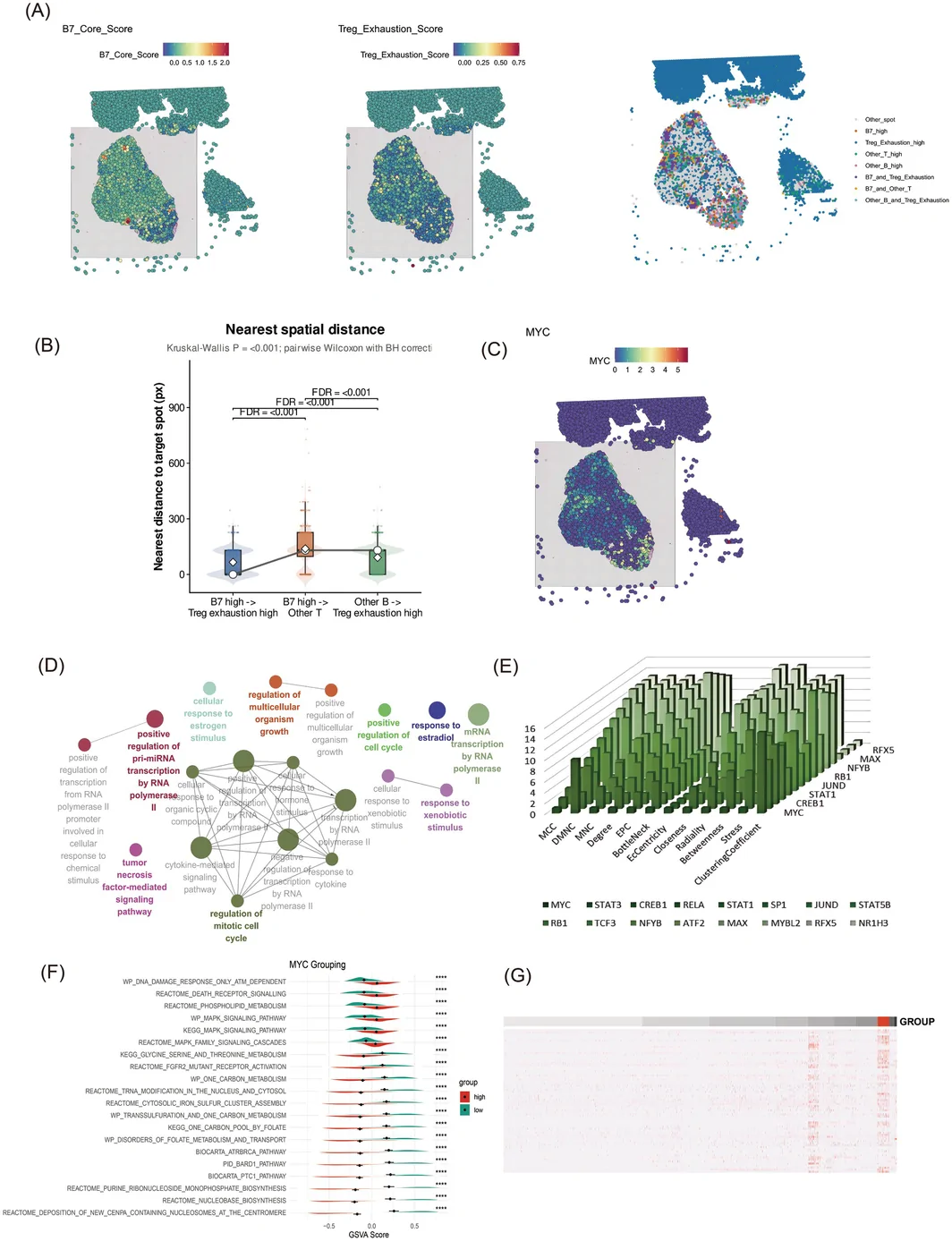
The B7 subpopulation is characterized by a MYC‐driven cell cycle program. (A) Spatial distribution of B7_Core_Score and Treg_Exhaustion_Score, together with categorical mapping of high‐score regions. (B) Nearest‐distance analysis showing significant spatial proximity between B7_high and Treg_Exhaustion_high spots. Statistical significance was assessed by Kruskal–Wallis test and pairwise Wilcoxon tests with BH correction. (C) Spatial expression pattern of MYC. (D) ClueGO enrichment of the key TFs confirms their role in cell cycle regulation. (E) PPI network identifying MYC as the top hub protein. (F) GSVA enrichment scores for hallmark pathways stratified by MYC expression. (G) Expression scores of a core cell cycle gene signature across B cell subpopulations, showing highest activity in B7. BH, Benjamini–Hochberg; GSVA, gene set variation analysis; PPI, protein–protein interaction; TF, transcription factor; Treg, regulatory T cell.

To investigate the spatial relationship between B7 core activity and Treg exhaustion, we visualized B7_Core_Score and Treg_Exhaustion_Score across tissue sections. Both scores were mainly enriched within tumor regions and showed partial spatial overlap (Figure 6A). Classification of spatial spots further revealed that B7_high, Treg_Exhaustion_high, and combined high‐score regions were primarily located within or near tumor areas, suggesting a spatial association between B7 core activity and the exhausted Treg phenotype. Nearest‐distance analysis supported this observation. B7_high spots were significantly closer to Treg_Exhaustion_high spots than expected from other spatial categories, with overall significance by Kruskal–Wallis test (*p* < 0.001) and significant pairwise comparisons after BH correction (FDR < 0.001) (Figure 6B). These findings indicate that B7 core‐active regions and Treg exhaustion‐enriched regions are not randomly distributed, but instead show significant spatial proximity. In addition, MYC expression was mainly enriched within tumor regions and partially overlapped with B7/Treg exhaustion‐associated areas, suggesting that MYC‐related transcriptional activity may be enhanced in this immunoregulatory spatial niche (Figure 6C). To further validate the regulatory hierarchy, we performed ClueGO enrichment analysis on the TFs identified in Figure 5. The resulting functional network (Figure 6D) corroborated their predominant enrichment in cell cycle‐related pathways, underscoring their central role in this biological process. Protein–protein interaction network analysis consistently identified MYC as the central hub TF (Figure 6E). GSVA associated MYC expression with cell cycle, ferroptosis, and MAPK pathways (Figure 6F). Cell cycle‐related gene expression was most prominent in the B7 subpopulation (Figure 6G), supporting its role via MYC‐driven dysregulation in the Class C TME.

To externally validate the spatial association described above, we analyzed an independent melanoma spatial transcriptomics dataset. Spatial spots were categorized according to B7_high, Treg_Exhaustion_high, and combined high‐score states. The spatial map showed local co‐distribution and partial overlap between B7_high and Treg_Exhaustion_high regions in melanoma tissue (Figure S6A). Nearest‐distance analysis further confirmed this pattern. B7_high spots were significantly closer to Treg_Exhaustion_high spots than to other T‐cell regions (*p* = 0.008). In contrast, the distances from B7_high to Other_T and from Other_B to Treg_Exhaustion_high showed only marginal significance (*p* = 0.064 for both comparisons) (Figure S6B). These results suggest that B7 core‐active regions tend to spatially neighbor Treg exhaustion‐enriched regions in the external melanoma cohort.

## 4 Discussion

This integrated multi‐omics study characterizes the MYCN‐driven “immune desert” TME in NB and reveals the critical interaction networks of T and B lymphocytes within it [60, 61]. Our findings also confirm the strong association between MYCN amplification and an immunosuppressive niche, consistent with prior reports [62]. Unsupervised clustering identified a Class C subtype defined by MYCN amplification, high mortality, and an “immune desert” signature, confirming prior reports.

The role of MYCN in immune evasion is multifaceted. Prior studies show MYCN downregulates MHC class I expression and suppresses the cGAS‐STING/interferon pathway, impairing immune sensing and T‐cell recruitment [61, 63]. It also reduces ligands for NK cell‐activating receptors and may skew macrophages towards a pro‐tumorigenic phenotype. Our data corroborate this broad immunosuppressive role, quantifying the deficient immune infiltration and impaired cancer‐immunity cycle in the Class C TME [64]. Mechanistically, while MYCN does not directly regulate NF‐κB, the elevated REL observed in T cells within MYCN‐amplified tumors suggests a non‐cell‐autonomous effect‐MYCN‐driven microenvironmental remodeling may indirectly activate NF‐κB signaling in infiltrating lymphocytes, a connection not previously established in NB.

A major methodological innovation of our study lies in the application of the Scissor algorithm to accurately project bulk‐level phenotypic classifications onto single‐cell resolution. This approach enables mapping of a bulk‐defined “immune desert” phenotype onto its single‐cell constituents, allowing precise identification of the lymphocyte subsets most affected by MYCN‐driven immunosuppression—a resolution unattainable by bulk deconvolution methods. This enabled deep immune cell profiling specifically within the molecular context of the Class C subtype [65, 66, 67]. We found that in this “immune desert,” T and B lymphocytes are not merely reduced in number but undergo profound functional reprogramming. This approach allowed us to move beyond generic immune scoring to a precise dissection of lymphocyte states within a defined high‐risk background.

In T lymphocytes, we identified EOMES and REL as key transcription factors in the Class C TME. EOMES, previously linked to T‐cell exhaustion [68, 69], is bioinformatically connected to the regulation of Transient Receptor Potential (TRP) channel genes, offering a novel mechanistic perspective. Concurrently, REL expression correlated with MYCN amplification and a shift towards T‐cell exhaustion. Functional experiments demonstrate that pharmacological inhibition of REL with IT‐901 enhances PBMC‐mediated tumor killing, supporting REL as a candidate target in the neuroblastoma TME.

Our study addresses a gap in NB research by profiling B‐cell subpopulations and identifying a pivotal B7 subset in the Class C TME. B7 exhibits strong cell‐cycle signaling and enhanced communication with exhausted T cells and Tregs. Ligand‐Receptor‐Transcription Factor (LR‐TF) analysis revealed an activated, MYC‐centered transcriptional network in B7 cells, suggesting MYC drives its dysregulation [70]. LR‐TF axis analysis positions B7 as a central “signal receiver” from exhausted T cells and Tregs—suggesting that B cells may play an active role in the immunosuppressive network, which has been relatively underexplored in NB. Spatial transcriptomics further corroborates this interaction: B7 core‐active regions and Treg/exhaustion‐enriched regions exhibit significant spatial proximity in NB tissue sections, and this pattern was externally validated in an independent melanoma cohort, supporting the relevance of B7‐Treg crosstalk beyond a single tumor type. This extends MYC's oncogenic role to immunomodulation within the TME [71].

Our integrated model suggests that T‐B cell interactions are crucial in maintaining the “immune desert.” The communication between the B7 subpopulation and exhausted/Treg cells creates a self‐reinforcing immunosuppressive circuit. This aligns with emerging research highlighting the importance of B‐cell and T‐cell crosstalk in cancer immunity [72]. For instance, Su et al. demonstrated that modulating T‐helper cells can alter the TME and improve therapy response in NB models, while other studies note the prognostic significance of specific T‐cell subsets and the regulation of B cells by γδ T cells in NB [73, 74, 75]. Our work contextualizes such interactions within the specific molecular framework of MYCN amplification.

This study has several limitations. First, the sample sizes for the single‐cell and proteomic analyses are modest, and independent multicenter cohorts are needed to validate the stability of the identified lymphocyte states. Second, although CCA/MNN integration was used to mitigate batch effects, residual technical variation and cross‐platform differences among public datasets cannot be completely excluded. Third, the EOMES‐TRP axis and B7/MYC hub are mainly supported by transcriptomic, network‐based, and spatial‐association evidence; direct perturbation experiments, B7‐focused co‐culture systems, ligand‐receptor blockade, and in vivo validation are needed to establish causality. Fourth, broader protein‐level validation beyond REL remains necessary. Finally, Visium spot‐level proximity supports spatial association but does not prove direct cell–cell contact or signaling, and the melanoma dataset should be interpreted only as external support for the spatial co‐localization pattern.

## 5 Conclusion

In summary, we propose a working model for the MYCN‐driven “immune desert”: MYCN‐amplified tumor cells create an immunosuppressive foundation. Within this environment, T lymphocytes skew towards exhaustion, with REL as a pharmacologically targetable regulator, while a specific B‐cell subset (B7) undergoes MYC‐driven dysregulation, becoming a hub for immunosuppressive communication with exhausted T cells and Tregs. Our integrated approach—combining single‐cell, spatial, and proteomic layers—identifies three interconnected therapeutic nodes: REL inhibition (supported by functional validation), the EOMES/TRP axis (a novel mechanistic hypothesis), and the B7‐MYC communication hub (highlighting underexplored B‐cell biology with spatial evidence of T‐B proximity). Each offers a distinct angle for immunotherapeutic intervention in this aggressive disease.

## Author Contributions

**Jia‐Jian Hu:** conceptualization, validation, investigation, writing – original draft preparation, writing – review and editing. **Wen‐Juan Kang:** conceptualization, methodology, data curation, writing – review and editing. **Jia‐Tong Hu:** validation, formal analysis, visualization. **Ye‐Xiong Li:** resources, funding acquisition. **Feng‐Ju Song:** resources, funding acquisition. **Min Deng:** resources, supervision, funding acquisition. All authors have read and agreed to the published version of the manuscript.

## Funding

This research was supported by the National Science Foundation of China (82272757 to M.D.); Noncommunicable Chronic Diseases‐National Science and Technology Major Project (2023ZD0502200 to M.D.); the Non‐profit Central Research Institute Fund of Chinese Academy of Medical Sciences (2021‐RC310‐013 to M.D.); the Chinese Academy of Medical Sciences Innovation Fund for Medical Sciences (2021‐I2M‐1‐067, 2023‐I2M‐2‐004 and 2024‐I2M‐ZD‐004 to M.D.); Beijing Hope Run Special Fund of Cancer Foundation of China (LC2021R02 to M.D.); National High Level Hospital Clinical Research Funding 80102022503 (to M.D.); Beijing Natural Science Foundation (7254409 to W.‐J.K.); Cancer Hospital of the Chinese Academy of Medical Sciences‐Shenzhen Hospital Collaboration Fund (CFA202201008 to M.D).

## Ethics Statement

This research was conducted in line with ethical standards and received approval from the Ethics Committee of the Cancer Hospital, Chinese Academy of Medical Sciences (Approval No. NCC2025C‐582), and was conducted in accordance with the Declaration of Helsinki.

## Conflicts of Interest

The authors declare no conflicts of interest.

## Supporting information


**Figure S1:** HALLMARK pathway enrichment in molecular subtypes. Heatmap of GSVA scores for selected pathways across subtypes A–D.

**Figure S2:** Quality control of the scRNA‐seq data. (A) Calculation of quality control (QC) metrics for each cell: number of unique genes detected (nFeature_RNA), total molecular counts (nCount_RNA), percentage of hemoglobin gene expression (percent_HB), and mitochondrial genome ratio (percent_mt). (B) Scatter plots validating the reliability of the post‐filtering results.

**Figure S3:** Single‐cell atlas of the neuroblastoma TME and projection of the bulk “immune desert” phenotype. (A) Quality control metrics for the scRNA‐seq dataset (9 samples, 32,363 cells). (B) tSNE visualization of 10 major annotated cell types in the integrated TME. (C) Cellular composition of the TME for NOC and Class C groups projected by the Scissor algorithm, showing a marked reduction in T and B lymphocytes in Class C. (D) tSNE of reclustered T lymphocytes (11,216 cells) annotated into 11 functional subpopulations. (E) tSNE of reclustered B lymphocytes (3445 cells) annotated into 10 functional subpopulations, including the B7 subset.

**Figure S4:** Analysis of the transcription factor EOMES. (A) Expression distribution of EOMES across T cells. (B) Predicted ceRNA network involving EOMES. (C) Top enriched pathway for associated microRNAs. (D) Correlations between TRP channel genes and a T cell exhaustion score. (E) Predicted EOMES binding motifs in TRP channel gene promoters.

**Figure S5:** Functional association of MYBL2 and TCF3. Heatmap of GSVA scores for biological processes in samples grouped by MYBL2 or TCF3 expression.

**Figure S6:** External validation of B7 core and Treg exhaustion spatial proximity in melanoma. (A) Spatial distribution of B7_high, Treg_Exhaustion_high, and combined high‐score spot categories in melanoma tissue. (B) Nearest‐distance analysis showing that B7_high spots are significantly closer to Treg_Exhaustion_high spots than to other T‐cell regions (*p* = 0.008).

## Data Availability

The data generated and/or analyzed during the current study are available from the corresponding author upon reasonable request.
